# Supplementary material for: HIVprotI: an integrated web based platform for prediction and design of HIV proteins inhibitors
Source: J Cheminform. 2018 Mar 9;10:12. doi: 10.1186/s13321-018-0266-y (PMC5845081; doi:10.1186/s13321-018-0266-y)
Supplement: Supplementary file 2 — Additional file 2. Source code of HIVProtI web server. [file 13321_2018_266_MOESM2_ESM.zip › HIVprotI_Source-code/hivprotI/weka-3-6-6/doc/weka/core/Capabilities.Capability.html]

Capabilities.Capability


---


|  |  |  |
| --- | --- | --- |
| **Overview** \| **Package** \| **Class** \| **Tree** \| **Deprecated** \| **Index** \| **Help** \| **Weka's home** | |  |
| **PREV CLASS**   **NEXT CLASS** | **FRAMES**    **NO FRAMES**     **All Classes** |  |
| SUMMARY: NESTED \| ENUM CONSTANTS \| FIELD \| METHOD | DETAIL: ENUM CONSTANTS \| FIELD \| METHOD |  |


---


## weka.core Enum Capabilities.Capability

```
java.lang.Object
  java.lang.Enum<Capabilities.Capability>
      weka.core.Capabilities.Capability
```

**All Implemented Interfaces:**: java.io.Serializable, java.lang.Comparable<Capabilities.Capability>

**Enclosing class:**: Capabilities

---

``` public static enum Capabilities.Capability extends java.lang.Enum<Capabilities.Capability> ```

enumeration of all capabilities

---

**Enum Constant Summary**

`BINARY_ATTRIBUTES`
  
          can handle binary attributes

`BINARY_CLASS`
  
          can handle binary classes

`DATE_ATTRIBUTES`
  
          can handle date attributes

`DATE_CLASS`
  
          can handle date classes

`EMPTY_NOMINAL_ATTRIBUTES`
  
          can handle empty nominal attributes

`EMPTY_NOMINAL_CLASS`
  
          can handle empty nominal classes

`MISSING_CLASS_VALUES`
  
          can handle missing values in class attribute

`MISSING_VALUES`
  
          can handle missing values in attributes

`NO_CLASS`
  
          can handle data without class attribute, eg clusterers

`NOMINAL_ATTRIBUTES`
  
          can handle nominal attributes

`NOMINAL_CLASS`
  
          can handle nominal classes

`NUMERIC_ATTRIBUTES`
  
          can handle numeric attributes

`NUMERIC_CLASS`
  
          can handle numeric classes

`ONLY_MULTIINSTANCE`
  
          can handle multi-instance data

`RELATIONAL_ATTRIBUTES`
  
          can handle relational attributes

`RELATIONAL_CLASS`
  
          can handle relational classes

`STRING_ATTRIBUTES`
  
          can handle string attributes

`STRING_CLASS`
  
          can handle string classes

`UNARY_ATTRIBUTES`
  
          can handle unary attributes

`UNARY_CLASS`
  
          can handle unary classes


| **Method Summary** | |
| --- | --- |
| `boolean` | `isAttribute()`             returns true if the capability is an attribute |
| `boolean` | `isAttributeCapability()`             returns true if the capability is an attribute capability |
| `boolean` | `isClass()`             returns true if the capability is a class |
| `boolean` | `isClassCapability()`             returns true if the capability is a other capability |
| `boolean` | `isOtherCapability()`             returns true if the capability is a class capability |
| `java.lang.String` | `toString()`             returns the display string of the capability |
| `static Capabilities.Capability` | `valueOf(java.lang.String name)`             Returns the enum constant of this type with the specified name. |
| `static Capabilities.Capability[]` | `values()`             Returns an array containing the constants of this enum type, in the order they are declared. |

**Methods inherited from class java.lang.Enum**

`compareTo, equals, getDeclaringClass, hashCode, name, ordinal, valueOf`

**Methods inherited from class java.lang.Object**

`getClass, notify, notifyAll, wait, wait, wait`

**Enum Constant Detail**

### NOMINAL\_ATTRIBUTES

```
public static final Capabilities.Capability NOMINAL_ATTRIBUTES
```

:   can handle nominal attributes

---


### BINARY\_ATTRIBUTES

```
public static final Capabilities.Capability BINARY_ATTRIBUTES
```

:   can handle binary attributes

---


### UNARY\_ATTRIBUTES

```
public static final Capabilities.Capability UNARY_ATTRIBUTES
```

:   can handle unary attributes

---


### EMPTY\_NOMINAL\_ATTRIBUTES

```
public static final Capabilities.Capability EMPTY_NOMINAL_ATTRIBUTES
```

:   can handle empty nominal attributes

---


### NUMERIC\_ATTRIBUTES

```
public static final Capabilities.Capability NUMERIC_ATTRIBUTES
```

:   can handle numeric attributes

---


### DATE\_ATTRIBUTES

```
public static final Capabilities.Capability DATE_ATTRIBUTES
```

:   can handle date attributes

---


### STRING\_ATTRIBUTES

```
public static final Capabilities.Capability STRING_ATTRIBUTES
```

:   can handle string attributes

---


### RELATIONAL\_ATTRIBUTES

```
public static final Capabilities.Capability RELATIONAL_ATTRIBUTES
```

:   can handle relational attributes

---


### MISSING\_VALUES

```
public static final Capabilities.Capability MISSING_VALUES
```

:   can handle missing values in attributes

---


### NO\_CLASS

```
public static final Capabilities.Capability NO_CLASS
```

:   can handle data without class attribute, eg clusterers

---


### NOMINAL\_CLASS

```
public static final Capabilities.Capability NOMINAL_CLASS
```

:   can handle nominal classes

---


### BINARY\_CLASS

```
public static final Capabilities.Capability BINARY_CLASS
```

:   can handle binary classes

---


### UNARY\_CLASS

```
public static final Capabilities.Capability UNARY_CLASS
```

:   can handle unary classes

---


### EMPTY\_NOMINAL\_CLASS

```
public static final Capabilities.Capability EMPTY_NOMINAL_CLASS
```

:   can handle empty nominal classes

---


### NUMERIC\_CLASS

```
public static final Capabilities.Capability NUMERIC_CLASS
```

:   can handle numeric classes

---


### DATE\_CLASS

```
public static final Capabilities.Capability DATE_CLASS
```

:   can handle date classes

---


### STRING\_CLASS

```
public static final Capabilities.Capability STRING_CLASS
```

:   can handle string classes

---


### RELATIONAL\_CLASS

```
public static final Capabilities.Capability RELATIONAL_CLASS
```

:   can handle relational classes

---


### MISSING\_CLASS\_VALUES

```
public static final Capabilities.Capability MISSING_CLASS_VALUES
```

:   can handle missing values in class attribute

---


### ONLY\_MULTIINSTANCE

```
public static final Capabilities.Capability ONLY_MULTIINSTANCE
```

:   can handle multi-instance data


**Method Detail**

### values

```
public static Capabilities.Capability[] values()
```

:   Returns an array containing the constants of this enum type, in
    the order they are declared. This method may be used to iterate
    over the constants as follows:

    ```
    for (Capabilities.Capability c : Capabilities.Capability.values())
        System.out.println(c);
    ```

    :   **Returns:**: an array containing the constants of this enum type, in the order they are declared

---


### valueOf

```
public static Capabilities.Capability valueOf(java.lang.String name)
```

:   Returns the enum constant of this type with the specified name.
    The string must match *exactly* an identifier used to declare an
    enum constant in this type. (Extraneous whitespace characters are
    not permitted.)

    :   **Parameters:**: `name` - the name of the enum constant to be returned. **Returns:**: the enum constant with the specified name **Throws:**: `java.lang.IllegalArgumentException` - if this enum type has no constant with the specified name: `java.lang.NullPointerException` - if the argument is null

---


### isAttribute

```
public boolean isAttribute()
```

:   returns true if the capability is an attribute

    :   **Returns:**: true if the capability is an attribute

---


### isClass

```
public boolean isClass()
```

:   returns true if the capability is a class

    :   **Returns:**: true if the capability is a class

---


### isAttributeCapability

```
public boolean isAttributeCapability()
```

:   returns true if the capability is an attribute capability

    :   **Returns:**: true if the capability is an attribute capability

---


### isOtherCapability

```
public boolean isOtherCapability()
```

:   returns true if the capability is a class capability

    :   **Returns:**: true if the capability is a class capability

---


### isClassCapability

```
public boolean isClassCapability()
```

:   returns true if the capability is a other capability

    :   **Returns:**: true if the capability is a other capability

---


### toString

```
public java.lang.String toString()
```

:   returns the display string of the capability

    :   **Overrides:**: `toString` in class `java.lang.Enum<Capabilities.Capability>`
    :   **Returns:**: the display string


---


|  |  |  |
| --- | --- | --- |
| **Overview** \| **Package** \| **Class** \| **Tree** \| **Deprecated** \| **Index** \| **Help** \| **Weka's home** | |  |
| **PREV CLASS**   **NEXT CLASS** | **FRAMES**    **NO FRAMES**     **All Classes** |  |
| SUMMARY: NESTED \| ENUM CONSTANTS \| FIELD \| METHOD | DETAIL: ENUM CONSTANTS \| FIELD \| METHOD |  |


---
